# The perception of time is dynamically interlocked with the facial muscle activity

**DOI:** 10.1038/s41598-019-55029-6

**Published:** 2019-12-10

**Authors:** Alexandre C. Fernandes, Teresa Garcia-Marques

**Affiliations:** ISPA – Instituto Universitário, William James Center for Research, Lisboa, Portugal

**Keywords:** Perception, Human behaviour

## Abstract

Time perception relies on the motor system. Involves core brain regions of this system, including those associated with feelings generated from sensorimotor states. Perceptual timing is also distorted when movement occurs during timing tasks, possibly by interfering with sensorimotor afferent feedback. However, it is unknown if the perception of time is an active process associated with specific patterns of muscle activity. We explored this idea based on the phenomenon of electromyographic gradients, which consists of the dynamic increase of muscle activity during cognitive tasks that require sustained attention, a critical function in perceptual timing. We aimed to determine whether facial muscle dynamic activity indexes the subjective representation of time. We asked participants to judge stimuli durations (varying in familiarity) while we monitored the time course of the activity of the zygomaticus-major and corrugator-supercilii muscles, both associated with cognitive and affective feelings. The dynamic electromyographic activity in corrugator-supercilii over time reflected objective time and this relationship predicted subjective judgments of duration. Furthermore, the zygomaticus-major muscle signaled the bias that familiarity introduces in duration judgments. This suggests that subjective duration could be an embodiment process based in motor information changing over time and their associated feelings.

## Introduction

Our ability to perceive time is very accurate, which is fundamental for many aspects of cognition that unfold in time, and for virtually all behaviors, from performing simple to complex actions^1^. However, contrasting with other basic physical dimensions (such as vision), the brain lacks a dedicated sensory system for processing time^2,3^.

Recent research suggests that explicit temporal processing is embedded in the motor system whose main functions are highly dependent on precise implicit timing^3–5^. Although a distributed neural network has been identified in general explicit timing^3,6^, only regions that are strongly implied in motor functions are consistently activated across *f*MRI studies^4^, the basal ganglia (BG), the cerebellum, the supplementary motor area (SMA) and the inferior frontal gyrus (IFG) that extends contiguously to the insular cortex (INS), as shown by the Wiener’ meta-analysis^5^ (but see also other meta-analyzes^7,8^). All these structures are involved in temporal and voluntary control of movement^4,9–11^ that is highly dependent on the sensorimotor feedback information (including somatic-kinesthetic afferent signals) in order to adjust and guide motor action^10–13^. However, it is unknown if the perception of time is an active process associated with specific patterns of muscle activity or its somatic-kinesthetic signals.

An already very old idea (going back to 1890) is that the sense of time is anchored in movement^14^ and arises as a feeling based on the related muscle tonus levels and contraction^15,16^. Of particular importance for this hypothesis is the central role of the insular cortex in motor and perceptual timing^5^, which is the primary brain region that enables higher-order representations of bodily states in the form of feelings^17^, including those associated with self-generated movements^12,13^. According to a recent proposal, the conscious representation of time is constructed in the insular cortex as the result of a temporal integration of these bodily feelings, which are generated over time from the interoceptive and proprioceptive systems^6,18^. This conjecture assumes the existence of a relationship between psychophysiological changes (over time) and time perception^19,20^.

In this paper, we address (for the first time) the hypothesis that temporal dynamics of muscle activity may index the subjective representation of duration. Generally, we explore how the coding of (spontaneous) motor activity supports perceptual timing. This idea was explored focusing on two different facial muscles that are linked to both affective and effort conscious feelings – the zygomaticus-major and corrugator-supercilii muscles –, and stems from the following observations.

Movement seems to shape the timing process^4,10^. Strong evidence of this comes from changes in temporal cognition in movement and motor system disorders^21^ like those observed in Parkinson^22,23^ and tremor^24,25^ patients. In normal individuals, movement during timing tasks seems to distort time perception as illustrated in the chronostasis illusion^26,27^, in the intentional binding illusion^28,29^ or in the prismatic adaptation procedure^30,31^. This effect appears to also occur in explicit perceptual timing tasks. For example, the duration of visual or tactil stimuli is compressed during hand movement^32,33^. These findings could imply that movement actively structures the perception of time even when it is supposedly not required, as in perceptual timing.

Conflicting activity in facial muscles have also been shown to interfere with time perception. Evidence indicates that when an automatic facial muscle’s response to emotional stimuli is inhibited (i.e., participants hold a pen in their mouth)^34^, the duration overestimation effect found in conditions of free facial expressions^35^ is cancelled out. This finding suggests that afferent feedback from facial muscles serve as an experiential source of information to estimate time^34^ as it occurs in other evaluative cognitive dimensions such as recognition and afective judgments^36,37^.

Interfering with the sensorimotor feedback in other body regions (e.g., tactile vibration stimulation of arms and hands) also seems to promote temporal distortions^33,38^. Sensorimotor integration could be essential to time perception^10,33^. In line with this embodied-cognition account, extensive research has shown unequivocally that emotions, and their associated body feelings, modulate time perception^39^, possibly also generated from proprioceptive-kinesthetic components of muscle activity related to emotional response^40,41^.

A leading proposal frames the representation of time in the isomorphic relationship between objective time and neural activity, which could be (or not) independent from peripheral physiology. A consistent finding across many studies has been a build-up of activity over time in brain structures of the timing network mostly in the insular cortex and SMA during perceptual timing (that peaks at the end of a given interval)^42–45^. This ramping of neural activity, which resembles a temporal cumulative process, has been attributed to the internal-clock like function that is the core of the dominant information-processing models of time perception^46,47^. However, given that a specific behavior outcome is expected, this neural ramping response has also been explained as a readiness state build-up in preparation for action^48^, or as an offline generation of a predictive model for action, which involves both motor and sensory components^10^.

In support of these hypotheses, several studies have found a continuous representation of stimulus duration in peripheral physiology that relates to action (i.e., cardiovascular metabolic support)^49^. Wittmann and colaborators^19,20^, for example, reported a linear increase of cardiac periods during the encoding of temporal intervals, and a correlation between the slope of this ramping activity and subjective duration estimates. In general, this cardiac response is considered a psychophysiological marker of attention^50,51^ and a perceptual orienting as a preparation for behavior-motor response^50,52^.

Attention is clearly relevant to prospective timing. Moreover, most of the brain structures of the timing network seem to be modulated by attentional processes^8^ or associated with attention control^53,54^, even during the time perception tasks. For instance, in several studies, duration encoding (i.e., climbing neural activity) in the SMA could not be disentangled from sustained attention (and mental effort) in timing tasks^55,56^. Estimating duration involves dynamic cognitive processes, namely sustained attention (to continuously track “temporal information”) and working memory processes (to continuously integrate and maintain a cumulative representation of duration over time), both of which requiring uninterrupted mental effort^57^.

Importantly, sustained attention (and effort) has been associated with an electromyographic (EMG) gradient^58^ during motor performance^59^ and mental tasks (without required motor output)^60,61^. It shows a steady, continuous rise in EMG activity that precipitates abruptly at the end of a task^58^. This EMG activity that only builds-up in tasks that require sustained attention can last from seconds to dozens of minutes, and typically reflects only muscle tension (or a covert action)^58^. These characteristics suggest a co-variation with physical time, similar to that of the climbing neural activity in SMA (and INS), and theoretically underlie the possibility of an effective motor component in “pure” perceptual timing.

Further relevant findings illustrate that EMG gradients are only found in muscles with high percentages of Type I slow-twitch extrafusal fibers, which are extremely slow-adapting^58,62^. This sets the stage for testing different types of muscles associated (also differently) with affective behavior and cognitive feelings. The documented myographic features are only present in a sub-group of facial muscles^60,61,63^. Specifically, the frontalis and corrugator-supercilii muscles (with a high percentage of Type I fibers) show uninterrupted EMG gradients, whereas the orbicularis-oculi and zygomaticus-major muscles (with a much lower percentage of Type I fibers) have failed to show EMG gradients in tasks requiring sustained attention and mental effort^60,61,63^. Of particular interest, the corrugator supercilii, a muscle responsible for frowning^64^, has also been associated with the expression of focused attention^60^, the expression of mental effort^61,63^ and the perception and feelings of effort^65^.

**Figure 1:**
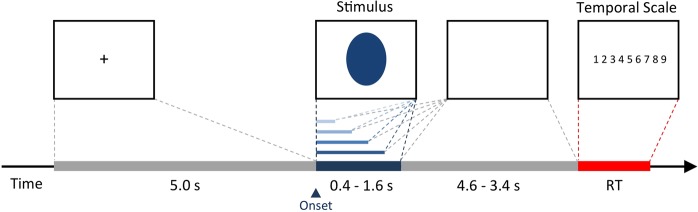
Schematic illustration of an experimental trial. After 5 s of a fixation signal (+), the stimulus (familiar or unfamiliar) appears in the center of the screen and lasts from 0.4 to 1.6 s. After stimulus offset, the screen remained blank until 5 s after the stimulus onset. The participant estimates the duration of the stimulus only when the 9-point temporal scale appears (1 = 0.4 s and 9 = 1.6 s).

The present study is the first investigation testing the dynamic relation between EMG activity and the subjective representation of time. To address this question, we measured muscle activity (with electromyography) in the corrugator-supercilii and zygomaticus-major muscles during the perceptual timing task (i.e., duration estimation of a visual stimulus) as a function of the objective duration of the stimulus (varying from 0.4 s to 1.6 s, see Fig. 1). We sought to find EMG gradients – a linear relationship between the objective duration of the stimulus and the duration and amplitude of muscle activation – and that this relationship be predictive of subjective duration. We expected this to occur for the corrugator-supercilii muscle but not for the zygomaticus-major muscle (basead on its physiomuscular properties). Additionally, we constinuously monitored the heart rate response (using the electrocardiography - ECG) as a control measure during the temporal task. This autonomic (re)activity is a relevant measure because indexes both attention and motor preparation for action^50–52^, and it is a consistent psychophysiological measure related with time perception^19,20^.

To strengthen our test, we added a variable (i.e., familiarity) to our study that we know to be able to both bias the perception of time and to promote a differential activation of the zygomatic-major and corrugator-supercilii muscles. This allows delimiting the expected effects. Stimuli familiarity is a factor known to positively bias the perceived duration of a stimulus^66,67^ and to promote a subjective experience of ease (vs. difficulty) that is compatible with the notion of a sense of reduced (vs. increased) mental effort^37,66^. Processing of an unfamiliar stimulus (i.e., a feeling of disfluency) is likely associated with an experience of greater difficulty in information processing, which is indexed by the increased activity of the corrugator-supercilii^61,63^. Thus, is expected a negative relationship between the bias promoted by familiarity and the activity of this muscle, an effect opposite to that expected in our main hypothesis (i.e., increasing corrugator activity as a function of objective and subjective duration). Given that the corrugator-supercilii is also associated with negative affect^64,68^, we could disantangle effort and sustained attention from negative feelings. Moreover, any impact of processing fluency (i.e., reduced mental effort) on the zygomaticus-major would not be related to mental effort, but rather to the hedonic positivity of familiar stimuli^69,70^ since that muscle is the one responsible for smiling and positive affect^64,68^. The participants’ task was to evaluate the duration of the stimuli – neutral faces –, which were either familiar to them (i.e., presented previous to the timing task) or not. We expected that stimulus familiarity would bias duration judgments and that over time this effect would also be indexed by muscle activity.

## Results

### Duration judgments

Analyses were performed by entering each dependent variable into a multifactorial analysis of variance (ANOVA), using stimuli duration, familiarity, and experiment block as the within-subject factors.

**Figure 2:**
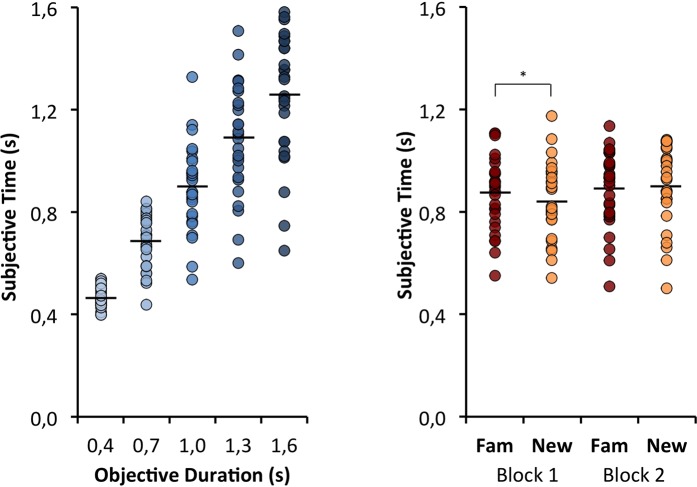
Duration estimation plots as a function of objective duration (left panel), and familiarity by trial block (right panel).

Figure 2 shows that participants were highly sensitive to the stimulus durations. The main effect of objective duration, F_(4,104)_ = 228.89, p < 0.0001, η² = 0.90, is almost perfectly represented by the time linear trend, t_(26)_ = 16.70, p < 0.0001, d = 3.21. None of the other factors directly or indirectly moderated this duration effect (all interactions had Fs < 1). Although there was no main effect of familiarity on duration estimates, F_(1,26)_ = 0.73, p = 0.399, η² = 0.03 (M_Fam_ = 0.879 s vs M_Unfam_ =  0.874 s), there was an interaction between familiarity and block, F_(1,26)_ = 5.12, p = 0.032, η² = 0.16. There was evidence of the expected effect of familiarity only in the first block, t_(26)_ = 2.23, p = 0.035, d = 0.43 (it dissipated in the second block t_(26)_ = −1.38, p = 0.18, d = 0.27, suggesting that the experience of familiarity changed through the task). We also found the expected main effect of block F_(1,26)_ = 7.77, p < 0.01, η² = 0.23, whichs indicates that durations were underestimated in the first block (0.857 s) compared with the second one (0.896 s).

### Psychophysiological analysis

For the sake of simplicity, we analyzed the physiologic data only for the experimental conditions in which an effect was clearly detected in participants’ duration estimates. Thus, the significance of the physiological effects was tested only for the first block in the case of familiarity, and for the whole experiment in the case of the stimuli objective duration. ANOVAs with repeated measures were performed separately for each of the factors, and the time course of the physiological response added as a second factor.

### Corrugator-supercilii activity and objective duration effects

**Figure 3:**
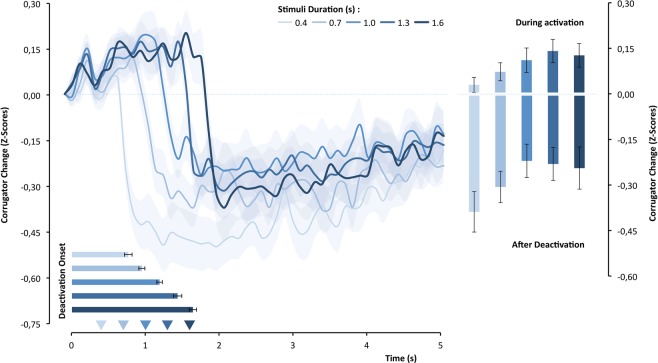
The standardized corrugator-supercilii activity over time as a function of objective duration of the stimulus. The superimposed bars represent the means of corrugator-supercilii activity during and after the activation period and the mean latency until muscle deactivation (latency).

Figure 3 shows that the corrugator-supercilii muscle changed as a function of the stimulus duration, F_(4,104)_ = 6.164, p < 0.0001, η² = 0.44. The upper panel of Fig. 3 illustrates, moreover, that there was clearly an initial period of the corrugator-supercilii activation followed by an abrupt deactivation. This difference in activity across time, F_(49,1274)_ = 20.22, p < 0.0001, η² = 0.19, was modulated by the objective duration of the stimulus, F_(196,5096) = _5.55, p < 0.0001, η² = 0.176. To understand how objective duration modulated the corrugator-supercilii activity, we analyzed its effects on deactivation latency and mean amplitude during and after deactivation. As hypothesized, the magnitude of CS activation differed significantly as a function of the objective duration of the stimulus, F_(4,104)_ = 3.02, p = 0.021, η² = 0.10, and had a positive linear component, t_(26) = _2.83, p < 0.01, d = 0.54. Statistically, this difference in corrugator-supercilii activity seemed to be maintained even after deactivation and stimuls offset, F_(4,104)_ = 3.04, p = 0.020, η² = 0.10, and also presented a linear trend at the limit of significance, t_(26)_ = 2.05, p = 0.0503, d = 0.395. As predicted, the deactivation latency covaries with the objective duration of the stimuli, F_(4,104)_ = 62.25, p < 0.0001, η² = 0.705. The accentuated linear trend was remarkable, t_(26)_ = 15.05, p < 0.0001, d = 2.90, as the lower graph of the top panel of Fig. 3 illustrates.

### Corrugator-supercilii activity mediation effects

**Figure 4:**
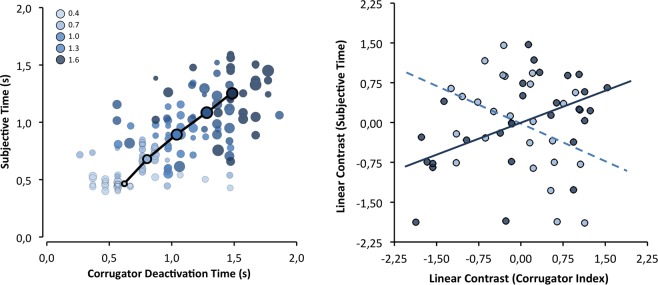
Left: group means (circles with black outlined) and individual means (light outline circles) of subjective duration as a function of corrugator-supercilii deactivation latency; circle diameter represents corrugator-supercilii activation amplitude until deactivation. Right: correlations (i.e., mediation effect) between subjective duration effect (linear contrast) with both CS deactivation latency effect (solid line) and activation amplitude effect (dashed line).

To test whether the corrugator-supercilii activity pattern mediated the effect of objective duration on subjective duration, we followed the within-mediation procedure of Judd and collaborators^71^ (with more than two levels). A regression analysis was conducted to compute separate linear trends (as a function of objective duration) for the corrugator-supercilii parameters and for subjective duration, using weights of −2, −1, 0, +1, and +2. As Fig. 4 shows, deactivation latency positively predicted the subjective duration estimation, β = 0.51, t_(25)_ = 2.95, p < 0.01. Unexpectedly, the amplitude of the corrugator activity did mediate subjective duration estimates, β = −0.44, t_(25)_ = −2.46, p = 0.02, but in the opposite direction. The same pattern was observed for the amplitude of corrugator-supercilii activity after deactivation, β = −0.42, t_(25)_ = −2.32, p = 0.03.

### Corrugator-supercilii activity and familiarity effects

No effects of familiarity were observed for corrugator-supercilii, F_(1,26)_ = 0.03, p = . 857, η² = 0.00, which suggested that familiarity did not affect mental effort (and negative affect). As expected, the analysis revealed the previously detected effect of time (course) in the corrugator-supercilii activity, F_(49,1274)_ = 13.95, p < 0.0001, η² = 0.35. This factor did not interact with familiarity either, F_(49,1274)_ = 0.76, p = 0.891, η² = 0.03.

### Zigomaticus-major activity and objective duration effects

The activity of the zigomaticus-major muscle changed over the time course, F_(49,1274)_ = 8.34, p < 0.0001, η² = 0.24. Yet, the activity of this muscle was not shown to be significantly modulated by the objective duration of the stimulus, F_(4,104)_ = 1.37, p = 0.250, η² = 0.05, showing as expected no clear sign of EMG gradients. However, stimulus objective duration seemed to impact the pattern with which the zigomaticus-major muscle was responded over time, F_(196,5096)_ = 1.84, p < 0.0001, η² = 0.07. Analysis of the first 2.5 s of zigomaticus-major activity after stimulus onset shows a tendency to decrease but it was not significant (a negative linear trend; t_(26)_ = −1.84, p = 0.083, d = 0.347).

### Zigomaticus-major activity and familiarity effects

A main effect of the familiarity factor, F_(1,26)_ = 5.57, p = 0.026, η² = 0.18, suggested a higher level of zigomaticus-major activation in the familiar conditions (−0.048) than in the unfamiliar conditions (−0.115). This effect was constant over the time course because of a non-significant interaction between these two factors, F_(49,1274)_ = 0.871, p = 0.723, η² = 0.03. As was previously detected, the variation in the zigomaticus-major over time was also significant, F_(49,1274)_ = 2.77, p = 0.0001, η² = 0.10.

### Zigomaticus-major activity mediation effects

**Figure 5:**
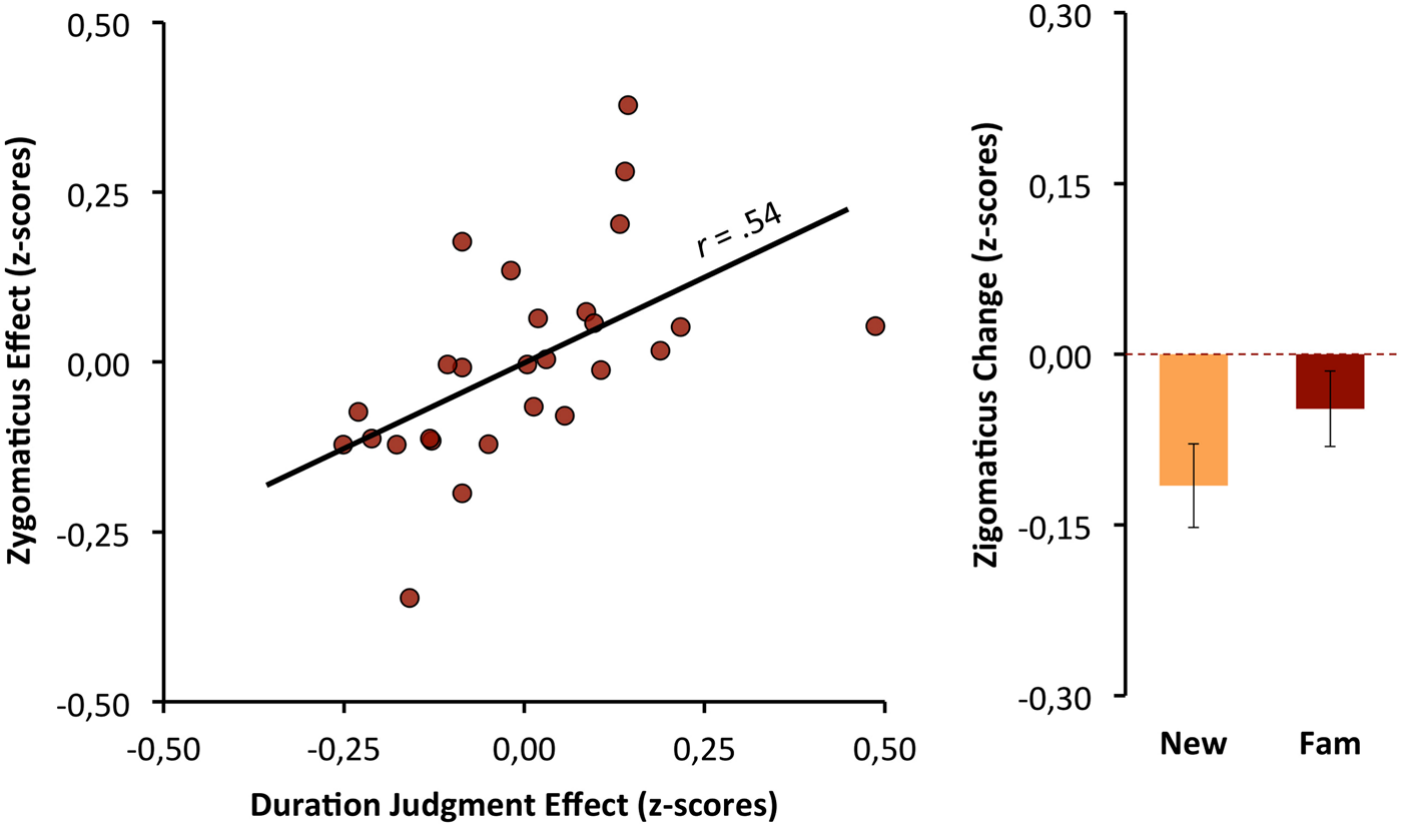
Left: correlation between subjective duration effect (familiar minus new stimuli) with zigomaticus-major effect (changes from baseline: familiar minus new stimuli). Right: group means of zigomaticus-major response for familiar and new stimuli.

To test whether the zigomaticus-major activity mediates the effect that familiarity had on subjective duration, we computed the relationship between the two effects, weighting the familiarity conditions as −1 and +1. The effects of familiarity on the zigomaticus-major were a good predictor of the bias that familiarity promotes for duration judgments, β = 0.54, t_(25)_ = 3.23, p < 0.01 (see Fig. 5).

### Heart-rate and objective duration effects

**Figure 6:**
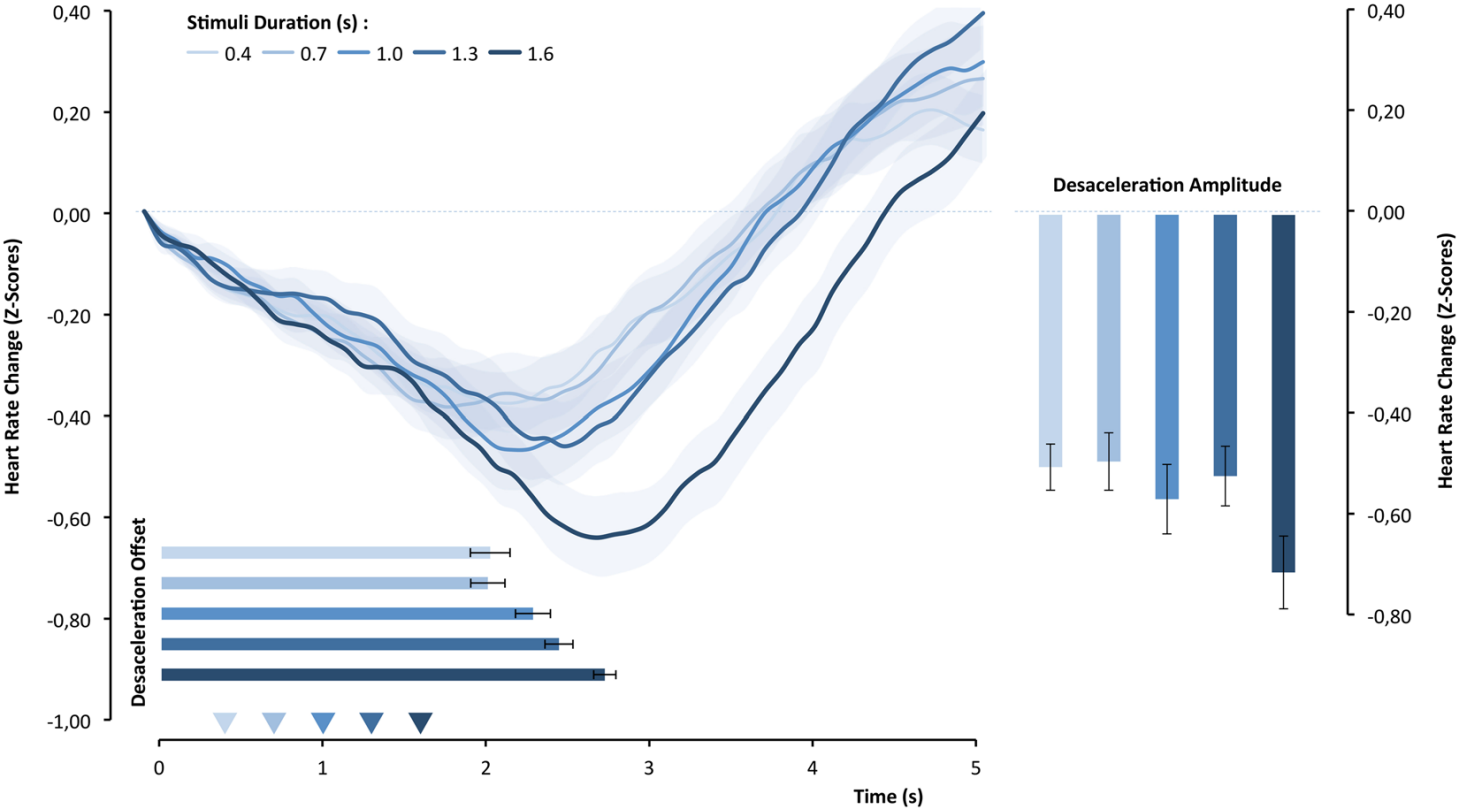
Standardized HR response over the time course as a function of objective duration of the stimulus. The superimposed bars show the means of HR deceleration amplitude and latency.

HR response to the face stimuli was deceleratory; it began shortly after stimulus onset and continued until reaching a minimum of several hundred milliseconds after stimulus offset. The 100 ms by 100 ms data are presented in Fig. 6 as a function of stimuli objective duration. HR changed significantly over the time course, F_(49,1274)_ = 41.89, p < 0.0001, η² = 0.617, and the trend analysis confirmed the reliability of the deceleration followed by an acceleratory period with a significant quadratic component, t_(26) = _4.45, p < 0.001, d = 0.857. The HR response itself seemed to be determined by the stimulus objective duration F_(1,26)_ = 3.23, p = 0.015, η² = 0.15, which was qualified by time-course, F_(196,5096)_ = 5.10, p < 0.0001, η² = 0.17. This qualification suggests that stimulus objective duration impacted not only the degree of HR response but also the previously observed pattern of that activation. To capture such influence, we calculated a set of new variables, the deceleration amplitude and the latency of the maximum deceleration, both of which occurred as a function of the objective duration of the stimulus (see Fig. 6). Stimuli duration promoted systematic positive related differences in the amplitude of HR deceleration, F_(4,104)_ = 3.55, p < 0.01, η² = 0.12, (the linear component explains the effect; t_(26)_ = 2.24, p = 0.034, d = 0.43), and a systematic decrease in the time required to achieve a minimum deceleration, F_(4,104)_ = 11.796, p < 0.0001, η² = 0.31, (the linear component by itself explains the effect, t_(26)_ = 5.85, p < 0.0001, d = 1.13).

### Heart-rate mediation effects

**Figure 7:**
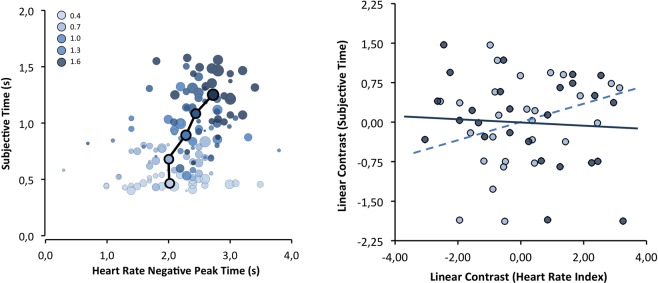
Left: group (circles outlined in black) and individual (light outline circles) means of subjective duration as a function of HR deceleration latency; circle diameter represents the amplitude of deceleration. Right: correlations (i.e., mediation effect) between subjective duration effect (linear contrast) with both HR deactivation latency effect (solid line) and HR amplitude effect (dashed line).

To test predictions from attention-based time perception theories, we examined whether differences in temporal judgments associated with stimulus duration could be predicted by the HR deceleration parameters (see Fig. 7). To directly test this, we followed the mediation approach of Judd and collaborators^71^ as we did for the corrugator-supercilii mediation effects; we used weights of −2, −1, 0, +1, and +2 to compute the HR parameters (amplitude and latency) and subjective duration as a function of objective duration. First, the within-subject mediation analysis revealed that deceleratory amplitude did not predict subjective duration, β = 0.28, t_(25)_ = 1.45, p = 0.159. Secondly, we tested whether the latency at the point of maximum HR deceleration could be the most relevant factor for predicting duration judgments. The relationship between the two trends, β = −0.08, t_(25)_ = −0.42, p = 0.68, suggested that this was not likely the case.

### Heart-rate and familiarity effects

HR activity was analyzed only for the first block, where we had previously detected an impact of familiarity in bias duration judgments. Using mean HR activity as a dependent measure in an ANOVA, and familiarity, objective duration and time course as the within-participant factors, no significant effects of the familiarity factor were revealed, F_(1,26)_ = 0.820, p = . 373, η² = 0.03. Only the previously noted impact of objective duration was detected, F_(49,1274)_ = 31.89, p < 0.0001, η² = 0.551, which was not moderated by the familiarity factor, F_(49,1274)_ = 0.318, p = . 999, η² = 0.01.

## Discussion

The main results of this study show that time perception is interlocked with the dynamic activity of the corrugator-supercilii. Namely, the relationship that perceptual timing has with the (spontaneous) motor output mediates subjective judgments of duration. These results provide the first empirical evidence of a motor component in (non-motor) perceptual timing, which is convergent with the suggestion that the central motor system is involved through action in building up a representation of time^4,10^.

Our findings reveal that both components that characterize the EMG gradients^58^ in the corrugator-supercilii – *muscle activation amplitude and the duration of that activation* – predicted the subjective duration judgments. This dynamic process of a build-up of activity over time is similar to those verified in critical brain regions for movement control during perceptual timing, particularly in the SMA and IFG-INS regions^42–45^. As for those known patterns of neural activity^6,42^, we can interpret this dynamic pattern of muscle activity as a temporal cumulative function representing subjective time. The main question that arises is how these two phenomena involving the motor system (at central and peripheral levels) could be interconected.

Primary, it is relevant to note that the climbing neural activity, mostly observed in the SMA, has been interpreted alternatively as a mere readiness potencial, off-line build-up as a preparation for action, a motor response associated with judgment, unrelated to time perception itself^48^. The present findings seem to discard this hypothesis in two ways. First, we expected a motor response only a few (3.4 to 4.6) seconds after the interval offset. Secondly, there was an actual muscle activation during the encoding of temporal information, which indicates that the motor activity may represent an active form of perception. Since interference with free/spontaneous movement distorts the perception of time as observed in previous work^26–29,32–34^, the present findings could imply that movement actively structures the perception of time^10^, even though no explicit movement was required during the perceptual timing task.

A process of active perception fits with embodied cognition theories inasmuch as bodily states generated through action can be used as an anchor for cognitive processing^13,36,37^. According to this view, we ground our perceptions (or reprocessing) of external events – *of any kind that unfold in time* – using our own sensorimotor system as an internal simulator^13^. As mentioned, the main brain regions implied in time perception, are also at the core of movement control functions^4,9–11^, which are achieved through the integration of (afferent) sensorimotor information, and the generation of top-down bodily predictions in order to adjust and guide motor actions^10–13^. It is argued that the predictive function of this system is used in a simulation (or emulation) mode as a way to (internally) reproduce external events, with varying degrees of re-enactement^13^. As an experiential route, this re-enactement process (i.e., motor behavior) facilitates the cognitive representation of specific patterns of sensorimotor changes across time^13^ and the generation of bodily feelings as the basis of affective and cognitive states associated with this behavior^36,37^. Accordingly, a simulation (or emulation) associated to a temporal pattern of sensorimotor changes, could be used to generate a feeling of time^6^. A recent conceptualization of temporal cognition sets the anterior insula as the basis for generating the sense of time achived through a process of temporal integration of bodily signals^6,18^. Strong support for this model is gathered in studies that show a build-up of neural activity in the anterior insula during perceptual timing^44,45^, a region that is associated with conscious awareness of bodily feelings including proprioceptive-kinesthetic awareness^17^. Our data is convergent with this assumption in the sense that the proprioceptive-kinesthetic feelings generated from the activation of the corrugator-supercilii (proportionally over time) can serve as information to estimating stimulus duration.

A linear progression is the simplest code to represent time, and it is in accordance with our results. However, one of the muscle activity components that we tested – *the amplitude component* – had a negative predictive value for subjective time, which represents an effect that decreases as objective time increases. This may be the result of a slowing of the increase in muscle activation amplitude over time – *a plateau effect of the EMG gradient* (see Fig. 3). This means that the temporal discrimination between longer objective durations based on this component becomes more difficult, in line with the scalar property^1^. This type of plateau effect is observed in SMA and INS^43,44^, and for this reason, it has been argued that this neuronal signal may not be an isomorphic representation of time^48^. Recent studies have found that neuronal coding of time in motor regions is relative (scalar) rather than absolute^72^. However, the EMG gradients in the corrugator and other facial (i.e., frontalis)^60,61,63^ and nonfacial muscles (i.e., forearm)^58,60^ can be (linearly) sustained over several minutes, suggesting that the peripheral motor components may have an isomorphic relationship with the subjective perception of time. On the other hand, the results of the present study may not be independent of a short duration range in the order of a few hundred milliseconds. It can emerge from an intrinsic relationship between the implicit perceptual timing and the precise motor timing in facial muscles in motor actions, such as those observed in speech or emotional facial mimickry^36,73^. Future studies should test other muscles using also longer objective durations to clarify this relationship.

Furthermore, the corrugator-supercilii facial muscle is strongly associated with affective and cognitive feelings that contributes to a miriade of judgments in other evaluative dimensions^36,37^. But future studies should address the interplay between central and peripheral components of the sensorimotor system and the mediation of proprioception-kinesthetic feelings generated from facial muscles in perceptual timing. In support of an embodiment account of time-perception, other research has found a linear relationship between interoception accuracy (i.e., heart beat perception), subjective duration and HR deceleration dynamic response in perceptual timing^19,20^, a cardiovascular response that is also associated with action preparation^50,52^. In the present study, although we found a relationship between objective duration and the HR deceleration response (amplitude and latency), it was not a predictor of subjective duration. This difference may be attributable to our shorter stimulus durations (0.4 to 1.6 s) compared with those of Wittmann and colleagues^19,20^ (8 to 20 s); electrocardiographic responses are slow (i.e., longer physiological response delay^64^) and may have persisted outside our temporal encoding window (e.g., latter components of the cardiac triphasic response^64,74^). Future studies should clarify these details.

Corrugator-supercilii and HR temporal dynamics have both been associated with processes of sustained attention (and effort)^50,60,61^. The generation of the EMG gradient verified in corrugator-supercilii may thus be caused by a sustained attentional process. Interestingly, however, this does not occur for HR. Again, the discrepancy arguably arises from the less reliability of HR activity in short durations. According to information-processing models of time perception, the timing process derives from the detection of temporal information through attention^46,47^. The research in time perception is amiss on the nature of this information. The present study suggests that this temporal information may be related to body feelings; specifically, those that emerge from proprioceptive-kinesthetic parameters. However, this poses a recursive problem of causality: attention causes the EMG gradient that becomes the focus of attention itself. An alternative explanation that must be considered is that attention is not the mechanism underlying the effect. Future studies should address the mechanisms associated with corrugator-supercilii activity and other muscles with similar myographic features, and account for sustained attention, proprioceptive-kinesthetic afferent signals, and brain top-down muscle control.

As expected the activity of zygomaticus-major muscle was unrelated with both objective duration and its relative subjective duration. This result is convergent with our expectation given its low percentage of Type I slow-twitch extrafusal fibers (and low adaptation) that are required to show EMG gradients under sustained attention conditions^58,62^.

Only the zigomaticus-major was sensitive to familiarity effects over duration judgments. Previous exposure promotes processing fluency and thus a subjective experience of ease that elicits judgments of positive affect^75,76^ indexed by the activation of the zigomaticus-major muscle^69,70^, which is responsible for smiling and positive affect^64,68^. However, the activation of the corrugator-supercilii seems more likely to signal disfluency since is associated with negative affect^60,77^, which was not promoted in our experiment. This pattern is also consistent with the fact that when the stimuli are fluent, frequently there is no observable corrugator-supercilii deactivation^69,70,77^. Thus, whereas the zigomaticus-major is related to the bias promoted by familiarity over duration judgment, corrugator-supercilii is related with duration judgment as a linear function of objective duration. This dissociation suggests that the former seems to be based on affective feelings, and the latter suggests that it is based on other non-affective types of feelings (i.e., proprioceptive-kinesthetic feelings associated with mental effort); although, in this case, it is not possible to totally discard an affective component since frowning integrates the facial motor output in many negative states and emotional reactions^64,68^. Either way, both effects are compatible with an embodiment account of time perception. Future studies should address the temporal bias of stimuli proprieties that are know to have a stronger impact in corrugator-supercilii activity; namely negative emotional stimuli that consistently (across studies) induce temporal overestimations^35,39^.

In conclusion, these results directly support an embodiment account for perceptual timing suggesting that duration judgments rely on bodily states including sensorimotor and affective systems^13,36,37^. They also define a process to be explored in greater detail given the consistent literature that involves the brain motor system as an amodal pathway that supports perceptual timing^4,5,10^. The idea that the sense of time could be based on somatic feelings^15^ related to movement^14^ and attention^16^ has old speculative roots, but now has a suggestive empirical support.

## Method

### Participants

Twenty-seven native Portuguese-speaking students (21 females) were recruited from various universities in Lisbon and were paid for their participation (10€), and provided written consent prior to the study. Participants were right-handed, had no history of attention disorder, and had normal or corrected-to-normal vision.

### Design, materials, and apparatus

The experiment was supported by a 2 × 2 × 5 repeated measure design with familiarity (prior exposure vs. first exposures), block (first vs. second block) and stimuli duration (0.4, 0.7, 1.0, 1.3 and 1.6 s) used as within-subject factors. The stimuli were presented using E-Prime 2.0 (Psychology Software Tools, Pittsburgh, PA) on a 19-inch monitor (100 Hz) with a resolution of 800 × 600, against a white background at a distance of 60 cm from the participant.

We used 80 neutral faces (all females) from the Lifespan Database of Adult Facial Stimuli^78^. For the training phase, we used 20 images of objects, controlled for levels of familiarity and valence (both neutral), from the Ficheiro de Imagens Multicategoriais (FIM) database^79^. The stimuli from both databases were re-sampled to have a visual angle of approximately 10°, converted to grayscale (16-bit), and matched for luminance and contrast.

### Facial EMG and ECG recording

Electrocardiography (ECG) and electromyographic (EMG) signals were collected using a Biopac MP100 system equipped with ECG100C and EMG100C amplifiers (Biopac Systems Inc., Goleta, CA). Both measures were amplified by a factor of 5,000 and sampled at 1,000 Hz frequency. Bipolar facial EMG (fEMG) signals were continuously recorded on the left corrugator-supercilii and zygomaticus-major muscles following a common pratice^68,70,77,80^, and using electrode placements recommended by Tassinari and colaborators^64^. This pratice is based on the typical symmetrical activation that is observed on both sides, and also because decreases the interference by the motor response (using the right hand). Moreover, it is unlikely that the unilateral measument of EMG has any impact on our data taking into account potential asymmetric correlations with different motor brain structures involved in perceptual timing (as a function of hemispheric lateralization). Taken together, the meta-analytic studies in perceptual timing^5,8^ reveal activations in critical structures (related with motor control) in both cerebral hemispheres. Two 4-mm Ag/AgCl electrodes were placed along the muscle fibers over each site with 1.5 cm between electrode centers. The skin surface at these sites was cleansed and gently abraded before placing the electrodes. fEMG signals were recorded with an online 10-Hz low-frequency cutoff filter, and a 500 Hz high-frequency cutoff filter. At recording, the ECG signal was bandpass filtered (0.5 and 35 Hz) and accessed using a standard 3-leads montage (Einthoven lead 2 configuration).

### Procedure

All procedures were approved by the ISPA – Instituto Universitário Ethics Review Board, and were performed in compliance with the principles of the Declaration of Helsinki. Participants arrived at the lab alone, and after signing the informed consent form, were asked to sit in front of a computer screen on an individual boot. The experimenter then took time to place the electrodes and explain to the participants that he would leave the room, but return by the time the experiment finished, and that all instructions would be presented on the computer screen. The instructions that were provided on the computer screen in the beginning of the experiment covered both the familiarization task and the temporal task. The whole experimental session took approximately 50 min.

#### Familiarization task

Subjects were first asked to complete a “familiarization” task that served to manipulate prior exposure to the target faces; they were told that they must be extremely attentive to each of the 40 target faces, which were presented for 1 s each with a 0.5 s inter-trial blank white screen.

#### Temporal task

Participants were first instructed to associate the extremes of a rating scale (1–9 points) with durations of 0.4 s and 1.6 s. Then, they performed 20 training trials with images of objects, in which they also evaluated intermediate durations of 0.7, 1.0, and 1.3 s, with the goal of “calibrating the use of the scale.” Each trial began with a cross (+) presented in the middle of the screen for 5.0 s followed by the object image (four randomly presented trials for each duration). After stimulus offset, the screen remained blank (i.e., white) until 5.0 s after the stimulus onset. This time lag was necessary to access the HR deceleration response and to avoid EMG contamination induced by hand movement in the behavioral response. Next, a prompt with the duration rating scale was displayed until a response was made; this was followed by a 0.5-s inter-trial interval. The same procedure was implemented for the test phase: in two different blocks, participants evaluated the duration of a neutral face that was either one of the 40 previously studied faces (i.e., a familiar photo) or one of 40 new faces. Four familiar photos and four new photos were presented for each of the five durations (4 targets × 5 durations). The 40 trials in each of the two blocks were presented randomly (a total of 80 trials). The participants rested for 3 minutes between the two experimental blocks (see Fig. 1).

#### Duration judgments

For the main analysis, the individual rating scale scores were converted to a temporal metric (i.e., *temporal value* = *[(score − 1) × 0.15] − 0.4*), in order to facilitate interpretation. These indexes were averaged for each experimental condition.

#### Facial EMG

Using Acqknowledge 4.4 software (Biopac Systems Inc., Goleta, CA), fEMG signals for corrugator-supercilii and zigomaticus-major muscles were visually inspected for noise, artifacts, and anomalous waveforms, and then filtered offline with a bandpass range of 20–400 Hz (the high-pass filtering at 20 Hz reduced blink, eye movement, and other low-frequency artifacts)^64^. A 50-Hz notch filter was also applied to reduce power line artifacts.

The filtered signals were then rectified, integrated, and smoothed over a 20-ms moving window. Next, 100 ms epochs, lasting from 1,000 ms pre-stimulus to 5,000 ms after onset, were averaged from the filtered signals, creating 60 distinct epochs. The data were then standardized (i.e., transformed to z-scores) within participants and muscle sites, attenuating the impact of highly reactive participants^64,81^. Trials with response errors, artifacts, or electromyographic activation exceeding five times the participant’s average standard deviation in each muscle within this time window were eliminated. Reported fEMG z-scores were expressed as changes from the average activity at baseline (400 ms to 0 ms), with values greater than 0 representing an increase over stimulus baseline. We used time windows under 500 ms^81^, and chose the 400-ms baseline window, following a similar strategy adopted by De Vries and collaborators^80^, as a way to minimize excessive artifacts. Additionally, we excluded trials in which the variation from baseline exceeded +/− 5 SDs to reduce the impact of extreme values. The mean for each design condition as a function of the 50 points in time was estimated for each of the muscles. According to the predictions of the corrugator-supercilii muscle responses (i.e., activation time and gradient), three indexes were calculated: (1) mean latency of the onset of deactivation as an index of activation duration, (2) mean amplitude during the activation period (until the onset of deactivation), and (3) mean amplitude during the period after deactivation (i.e., returning to baseline). The deactivation latency was defined as the point at which there was a continuous decrease in activity lasting at least 400 ms, which was operationalized as the largest difference between four points (of 100 ms) in the time-course within the 5 s window following the stimulus onset. As an index of the EMG gradient, we opted for the average corrugator-supercilii activation amplitude until deactivation onset rather than the amplitude peak because there is substantial variability between the time-course epochs. As predicted, the zigomaticus-major muscle did not show a pattern compatible with an EMG gradient – even a negative one – and showed greater variability, which impeded the calculation of any of the previous indexes.

#### Heart rate

After the exclusion of artifacts, heartbeats per minute (bpm) were derived offline from the ECG signals through an algorithm that computed the interval time between successive R-waves. To standardize and baseline correct, we applied the same procedure that we applied to the EMG data. Additionally, for the mean of each condition for the 50 points of HR time-course response, we calculated the deceleration amplitude of the HR (as an index of attentional orientation) and the latency until maximum deceleration (as an exploratory index of attentional dependence related to the duration of the stimulus). Following Bradley and collaborators^74^, we calculated the amplitude of HR deceleration as the difference between the maximum and minimum values within the 4 s window (from the onset of the stimulus).
